# Tickborne Pathogen Detection, Western Siberia, Russia

**DOI:** 10.3201/eid1111.041195

**Published:** 2005-11

**Authors:** Vera A. Rar, Natalia V. Fomenko, Andrey K. Dobrotvorsky, Natalya N. Livanova, Svetlana A. Rudakova, Evgeniy G. Fedorov, Vadim B. Astanin, Olga V. Morozova

**Affiliations:** * Institute of Chemical Biology and Fundamental Medicine, Novosibirsk, Russia; † Institute of Systematics and Ecology of Animals, Novosibirsk, Russia; ‡ Omsk Research Institute of Natural Focus Infections, Omsk, Russia; § Center of Epidemiological Control of Novosibirsk Region, Novosibirsk, Russia

**Keywords:** Ixodes persulcatus, Dermacentor reticulatus, Borrelia burgdorferi sensu lato, Anaplasma phagocytophilum, Ehrlichia muris, Bartonella, Babesia canis canis, nested PCR, sequencing, research

## Abstract

*Ixodes persulcatus* (n = 125) and *Dermacentor reticulatus* (n = 84) ticks from Western Siberia, Russia, were tested for infection with *Borrelia*, *Anaplasma*/*Ehrlichia*, *Bartonella*, and *Babesia* spp. by using nested polymerase chain reaction assays with subsequent sequencing. *I. persulcatus* ticks were infected with *Borrelia burgdorferi* sensu lato (37.6% ± 4.3% [standard deviation]), *Anaplasma phagocytophilum* (2.4% ± 1.4%), *Ehrlichia muris* (8.8% ± 2.5%), and *Bartonella* spp. (37.6% ± 4.3%). *D. reticulatus* ticks contained DNA of *B. burgdorferi* sensu lato (3.6% ± 2.0%), *Bartonella* spp. (21.4% ± 4.5%), and *Babesia canis canis* (3.6% ± 2.0%). *Borrelia garinii*, *Borrelia afzelii*, and their mixed infections were observed among *I. persulcatus*, whereas *B. garinii* NT29 DNA was seen in samples from *D. reticulatus*. Among the *I. persulcatus* ticks studied, no *Babesia* spp. were observed, whereas *B. canis canis* was the single subspecies found in *D. reticulatus*.

## Abstract 2

*Ixodes* and *Dermacentor* ticks harbor *Borrelia*, *Anaplasma*/*Ehrlichia*, *Bartonella*, and *Babesia* species.

Ticks are second only to mosquitoes as vectors of bacterial, viral, and protozoan agents (*1*). Among tickborne bacteria, extracellular spirochetes of the genus *Borrelia* are widely spread and most studied. Some of these, those that belong to the *Borrelia burgdorferi* sensu lato complex, are causative agents of Lyme borreliosis (*1**,**2*). Other known pathogenic bacteria transmitted by ticks are intracellular alpha proteobacteria, which includes the families *Anaplasmataceae*, *Bartonellaceae*, and *Rickettsiaceae* (*3*). Members of the genera *Anaplasma* and *Ehrlichia*, from the family *Anaplasmataceae*, infect mainly monocytes and granulocytes and cause human and animal anaplasmoses and ehrlichioses (*4*). Bacteria of the genus *Bartonella* infect erythrocytes and endothelial cells. Different species of *Bartonella* are the etiologic agents of cat-scratch disease, trench fever, and Carrión disease (*5**,**6*). The tickborne protozoa of the genus *Babesia* reproduce in erythrocytes, thus causing babesiosis among humans as well as wild and domestic animals (*7*).

Prevalent tick species for forest-steppe zones of Western Siberia, Russia, are taiga ticks (*Ixodes persulcatus* Schulze) and meadow ticks (*Dermacentor reticulatus*) (Acarina: Ixodidae) (*1**,**2**,**8*). They parasitize many ground-foraging bird species and virtually all the terrestrial mammals (*8*); both species are able to feed on humans (*8**,**9*) and transmit different tickborne infections (*2*).

The *I. persulcatus* habitat is the southern part of the forest zone of Eurasia (*1**,**2*). Until 1987, only tickborne encephalitis virus was thought to be associated with taiga ticks, but extensive studies have shown their competence in the transmission of pathogenic spirochetes, *Borrelia garinii* and *Borrelia afzelii* (*2*). Recently, *Anaplasma*/*Ehrlichia* was found in *I. persulcatus* ticks (*10*). The main *Ehrlichia* species found in *I. persulcatus* ticks is a recently characterized species, *Ehrlichia muris*, isolated from a wild mouse in Japan (*11**–**13*). The etiologic agent of human granulocytic anaplasmosis, *Anaplasma phagocytophilum*, has also been found in *I. persulcatus* ticks (*14**–**16*). Infection of ixodid ticks with *Bartonella* spp. has recently been described in the United States (*17*), Europe (*18**,**19*), and Western Siberia (*20*). Infection of *I. persulcatus* with *Babesia microti* pathogenic for immunocompromised humans has been shown by polymerase chain reaction (PCR) with genus- and species-specific primers (*21*), but nucleotide sequences of the specific PCR products remain unknown.

The second tick species, *D. reticulatus*, inhabits meadows and pastures (*2*), as well as near suburban areas from Europe to central Asia, but not taiga and dry steppes (*22*). *D. reticulatus* is well known as the vector of a canine pathogen, *Babesia canis canis* (*23*); *Rickettsia* spp (*13*), *Francisella tularensis*, and *Coxiella burnetii* were also found in this tick species (*1*). *Borrelia* spp. was also detected in different *Dermacentor* species, including *D. reticulatus*, by means of PCR (*9*) and an indirect immunofluorescence assay (*24*). Infection of *D. reticulatus* with *Anaplasma*/*Ehrlichia* and *Bartonella* species was previously unknown in spite of the detection of bacterial DNA in other *Dermacentor* species (*17**,**25*). Little or nothing was known of the genetic variability of the tickborne pathogens in ixodid ticks from Western Siberia (*2*); consequently, the aim of the present study was to study prevalence and genetic diversity of *Borrelia*, *Anaplasma*/*Ehrlichia*, *Bartonella*, and *Babesia* among *I. persulcatus* and *D. reticulatus* ticks in Western Siberia, Russia.

## Materials and Methods

**Figure 1:**
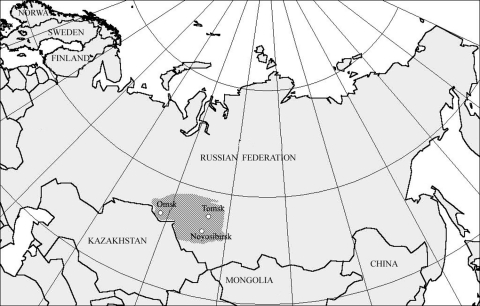
Areas where ticks were collected. Omsk, Tomsk, and Novosibirsk regions are shaded.

Unfed adult *I. persulcatus* ticks were collected by flagging of lower vegetation in different suburban places of mixed aspen-birch and pine forests of Novosibirsk (55°N, 83°E) (115 ticks) and Tomsk regions (56°N, 85°E) (12 ticks) (Figure 1) in May and June of 2003 and 2004. Questing imago of *D. reticulatus* ticks were collected by flagging in different locations of river valley and forest-steppe zones of Novosibirsk (72 ticks) and Omsk regions (55°N, 73°E) (15 ticks) from May to June of 2003 and 2004 (Figure 1). Nucleic acids were isolated by lysis of 127 individual *I. persulcatus* and 87 *D. reticulatus* ticks in guanidine thiocyanate followed by deproteinization with phenol-chloroform and precipitation with isopropanol.

### PCR Assay

To prevent contamination, we performed DNA isolation, PCR master mix assembly, and amplifications in separate rooms. Aerosol-free pipette tips were also used at each stage. We included negative control reactions with bidistillated water in each experiment at both steps of nested PCR. All reactions were performed in 20 μL reaction mixture containing 67 mmol/L Tris-HCl (pH 8.9), 16.6 mmol/L (NH_4_)_2_SO_4_, 2 mmol/L MgCl_2_, 0.01% Tween-20, 200 μmol/L each dNTP, 5% glycerol, 0.5 μmol/L specific primers, 2 U Taq DNA polymerase (Institute of Chemical Biology and Fundamental Medicine, Siberian Branch of the Russian Academy of Sciences), and 2 μL tested DNA. Amplification was performed in a Tercic Thermal Cycler (DNA Technology, Moscow, Russia). For the inner reactions, 2 μL of the outer PCR products were added into the reaction mixture. PCR fragments were visualized under UV irradiation after electrophoresis in agarose gels containing ethidium bromide. To control DNA isolation from ticks, we performed PCR on an aliquot of purified DNA with the following universal primers targeted to 18S rRNA gene: forward 5´-AACCTGGTTGATCCTGCCAGTAGTCAT-3´ and reverse 5´-GAATGATCCTTCCGCAGGTTCACCTAC-3´ (26).

To detect tickborne infectious agents, we used nested PCR with specific primers both previously described (*6**,**27**,**28*) and designed in our study. Multiple sequence alignment of nucleotide sequences available in GenBank (http://www.ncbi.nlm.nih.gov) for each tickborne pathogen was performed by using ClustalW (http://www.ebi.ac.uk/clustalw/) (*29*). The desired specificity of selected primers and absence of cross-reactions were confirmed by BlastN homology search (http://www.ncbi.nlm.nih.gov/BLAST/). Formation of intra- and intermolecular primer dimers was reduced by using OLIGOS (http://www.basic.northwestern.edu/biotools/oligocalc.html).

### PCR Detection of *Borrelia*-specific DNA

*B. burgdorferi* sensu lato DNA was detected by means of nested PCR with primers specific to conservative regions of 5S and 23S rRNA genes to amplify variable intergenic spacer (*27*). Primers for outer PCR were designed by aligning 5 GenBank nucleotide sequences of rRNA gene clusters for 3 species belonging to *B. burgdorferi* sensu lato complex, including *B. burgdorferi* sensu stricto, *B. garinii*, and *Borrelia lusitaniae*. Other criteria for primer design included the absence of possible cross-reactions with other genera. Outer reactions were performed with NC1 (5´-CCTGTTATCATTCCGAACACAG-3´) and NC2 (5´-TACTCCATTCGGTAATCTTGGG-3´) primers (35 cycles of 60 s at 94°C, 30 s at 58°C, and 30 s at 72°C). Inner reactions were carried out as previously described (*27*). DNA isolated from *B. burgdorferi* sensu stricto (strain B31), *B. afzelii* (strain Ip-21), and *B. garinii* (strains T6, 2, and 12) was used as positive control. Molecular typing of the PCR-positive samples was performed by using sequencing and restriction fragment length polymorphism analysis (*27*) by hydrolysis of the PCR products with the *Tru9*I restriction endonuclease (SibEnzyme, Novosibirsk, Russia) (isoschizomer *MseI*) with subsequent electrophoresis in 15% polyacrylamide gel.

### PCR Detection of *Anaplasma*- and *Ehrlichia*-specific DNA

For *Anaplasma*/*Ehrlichia* detection, specific primers were designed by comparing 15 nucleotide sequences of 16S rRNA gene from 12 species (*A. phagocytophilum*, *A. bovis*, *A. platys*, *A. centrale*, *A. marginale*, *E. muris*, *E. ruminantium*, *E. ewingii*, *E. chaffeensis*, *E. canis*, *Wolbachia pipientis*, *Rickettsia rickettsii*). Primers EHR1, EHR2, and EHR3 were identical to the 16S rRNA gene nucleotide sequences of *Anaplasma*/*Ehrlichia* species but differed from *W. pipientis* sequence and markedly distinguished from the sequence of *R. rickettsia*. Outer reactions were performed by using EHR1 (forward, 5´-GAACGAACGCTGGCGGCAAGC-3´) and EHR2 (reverse, 5´-AGTA(T/C)CG(A/G)ACCAGATAGCCGC-3´) primers and inner reactions with EHR3 (forward, 5´-TGCATAGGAATCTACCTAGTAG-3´) and EHR2 primers (35 cycles of 1 min at 94°C, 1 min at 55°C, and 1 min at 72°C). DNA isolated from *A. phagocytophilum* and *A. marginale* from spleens of ill cattle were used as positive controls. For PCR-positive samples, nested reactions with *A. phagocytophilum*–specific primers HGE1 (forward, 5´-CGGATTATTCTTTATAGCTTGC-3´) and HGE2 (reverse, 5´-CTTACCGAACCGCCTACATG-3´) were carried out in the same conditions.

### PCR Detection of *Bartonella*-specific DNA

For *Bartonella* DNA detection, nested PCR with primers corresponding to *groEL* gene (*6*) was used. For outer reactions, primers BH1 (forward, 5´-GAAGAAACAACTTCTGACTATG-3´) and BH4 (reverse, 5´-CGCACAACCTTCACAGGATC-3´) were designed by aligning 31 nucleotide sequences of 13 *Bartonella* species, including *Bartonella henselae*, *Bartonella quintana*, *Bartonella alsatica*, *Bartonella birtlesii*, *Bartonella bacilliformis*, *Bartonella capreoli*, *Bartonella doshiae*, *Bartonella grahamii*, *Bartonella koehlerae*, *Bartonella schoenbuchensis*, *Bartonella taylorii*, *Bartonella tribocorum*, and *Bartonella vinsonii*. The outer reactions were carried out in 45 cycles of 30 s at 94°C, 30 s at 55°C, and 45 s at 72°C. Both primer structures (HSP1, HSP2, and HSP4) and PCR conditions (45 cycles of 30s at 94°C, 30 s at 58°C, and 45 s at 72°C) for inner seminested reactions in the presence of 3 primers were identical to those previously described (*6*). Full-length genomic DNA samples isolated from *B. henselae* and *B. quintana* were used as positive controls.

### PCR Detection of *Babesia*-specific DNA

*Babesia* DNA was detected by means of nested PCR with primers specific to the 18S rRNA gene. Specific primer PiroA had been previously described (*28*). Other *Piroplasmida*-specific primers were designed by comparing the 18S rRNA gene nucleotide sequences of 11 species (*B. canis canis*, *Babesia canis vogeli*, *Babesia canis rossi*, *Babesia odocoilei*, *Babesia divergens*, *Babesia caballi*, *Babesia gibsoni*, *B. microti*, *Theileria parva*, *T. equi*, *Plasmodium falciparum*). The chosen primers corresponded to the sequences of most *Piroplasmida* species (including those listed above) but significantly differed from those of *P. falciparum*. Outer reactions were performed with BS1 (forward, 5´-GACGGTAGGGTATTGGCCT-3´) and PiroC (reverse, 5´-CCAACAAAATAGAACCAAAGTCCTAC-3´) primers (36 cycles of 1 min at 94°C, 1 min at 59°C, and 1 min at 72°C) and inner reactions with PiroA (forward, 5´-ATTACCCAATCCTGACACAGGG-3´) according to Armstrong et al. (*28*) (with the single nucleotide transition A→T at position 2 from the 5´ end of the primer) and PiroC primers (36 cycles of 1 min at 94°C, 1 min at 57°C, and 1 min at 72°C). For subsequent sequencing, the 1,304-bp fragment was synthesized in PCR with BS1 and BS2 (reverse, 5´-ATTCACCGGATCACTCGATC-3´) primers (40 cycles of 1 min at 94°C, 1 min at 57°C, and 2 min at 72°C).

*B. canis canis* DNA isolated from blood samples of a dog with clinical signs of babesiosis confirmed by microscopic examination (GenBank accession no. AY527064) and *B. microti* DNA from *Clethrionomys rutilus* blood (AY943958) (V. Rar, unpub. data) were used as positive controls.

### Sequencing of PCR Products

The PCR products were purified after gel electrophoresis in 1.5%–2% agarose gels with GFX Columns (Amersham Biosciences, Piscataway, NJ, USA) according to the manufacturer's instructions. Nucleotide sequences of the PCR products were determined by using BigDye Terminator Cycle Sequencing Kit and the ABI PRISM 310 Genetic Analyzer (Applied Biosystems, Foster City, CA, USA) at the DNA Sequencing Centre of the Siberian Branch of the Russian Academy of Sciences, Novosibirsk, Russia. For initial species identification, the nested PCR products were sequenced in 1 direction. Detailed confirmation for each genetic group was performed by sequencing with forward and reverse outer or inner primers as needed.

**Figure 2:**
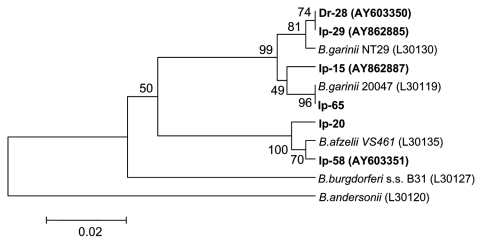
Phylogenetic tree based on the Borrelia burgdorferi sensu lato 5S-23S rRNA intergenic spacer fragment sequences. Scale bar indicates an evolutionary distance of 0.02 nucleotides per position in the sequence. Borrelia andersonii was used as outgroup. Numbers above the branches indicate bootstrap support indexes. Samples isolated from Ixodes persulcatus (Ip) and Dermacentor reticulatus (Dr) in this research are in boldface.

**Figure 3:**
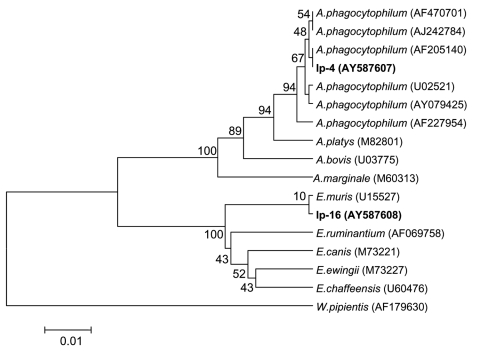
Phylogenetic tree based on the Anaplasma/Ehrlichia 16S rRNA gene fragment sequences. Scale bar indicates an evolutionary distance of 0.01 nucleotides per position in the sequence. Wolbachia pipientis was used as outgroup. Numbers above the branches indicate bootstrap support indexes. Samples from Ixodes persulcatus (Ip-4 and Ip-16) from this study are in boldface.

**Figure 4:**
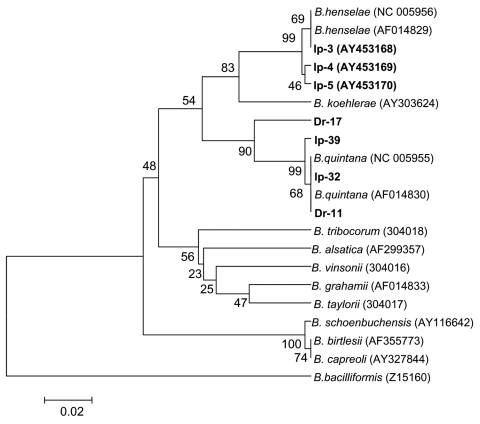
Phylogenetic tree based on the Bartonella groEL gene fragment sequences. Scale bar indicates an evolutionary distance of 0.02 nucleotides per position in the sequence. Bartonella bacilliformis was used as outgroup. Numbers above the branches indicate bootstrap support indexes. Samples from Ixodes persulcatus (Ip) and Dermacentor reticulatus (Dr) from this study are in boldface.

**Figure 5:**
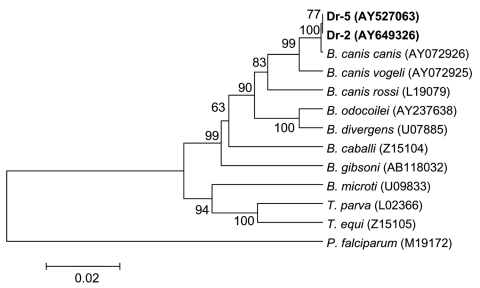
Phylogenetic tree based on the Babesia 18S rRNA gene fragment sequences. Scale bar indicates an evolutionary distance of 0.02 nucleotides per position in the sequence. Plasmodium falciparum was used as outgroup. Numbers above the branches indicate bootstrap support indexes. Samples from Dermacentor reticulatus (Dr-2 and Dr-5) from this study are in boldface.

Nucleotide sequences of PCR products determined in this study were analyzed by BlastN and aligned with ClustalW (*29*). Phylogenetic analysis was performed with MEGA 3.0 software (*30*). We used the unweighted pair-group method with arithmetic mean (UPGMA) and neighbor-joining algorithms with the Kimura 2-parameter model to generate the distance matrix as well as maximum parsimony and minimal evolution with a heuristic search. Bootstrap analysis was performed with 1,000 replications. GenBank accession numbers for the sequences used in the phylogenetic analysis are shown in Figures 2–5.

The nucleotide sequences determined in this study were deposited in GenBank under the following accession numbers: *B. garinii*, AY603350, AY862887, AY862885; *B. afzelii*, AY603351; *A. phagocytophilum*, AY587607; *E. muris*, AY587608; *B. henselae*, AY453166–AY453170; *B. canis canis*, AY527063, AY649326.

## Results

Infection of *I. persulcatus* and *D. reticulatus* with 3 bacterial and 1 protozoan tickborne pathogens in Western Siberia, Russia were studied by nested PCR with genus-specific primers. To control DNA suitability for PCR analysis, we amplified the 18S rRNA gene in 125 of the 127 *I. persulcatus* samples tested and in 84 of the 87 *D. reticulatus* ticks studied. Therefore, the 5 samples in which we were unable to amplify tick DNA were excluded from further analysis. Both tick species contained *Borrelia* and *Bartonella* DNA, whereas *Anaplasma*/*Ehrlichia* DNA was detected only in *I. persulcatus*, and *Babesia* DNA was detected only in *D. reticulatus* ticks (Table).

**Table:** Prevalence of tickborne infectious agents in ticks in Western Siberia, Russia, 2003–2004

Pathogen	Prevalence (% ± SD)*	
	*Ixodes persulcatus*	*Dermacentor reticulatus*
*Borrelia* spp.	37.6 ± 4.3	3.6 ± 2.0
*Borrelia garinii* NT29	18.4 ± 3.5	3.6 ± 2.0
*B. garinii* 20047	8.8 ± 2.5	0
*Borrelia afzelii*	8.8 ± 2.5	0
Mixed *B. garinii* NT29 + *B. afzelii*	1.6 ± 1.1	0
*Anaplasma*/*Ehrlichia* spp.	11.2 ± 2.8	0
*Ehrlichia muris*	8.8 ± 2.5	0
*Anaplasma phagocytophilum*	2.4 ± 1.4	0
*Bartonella* spp.	37.6 ± 4.3	21.4 ± 4.5
*Babesia* spp.	0	3.6 ± 2.0
*Babesia canis canis*	0	3.6 ± 2.0

*SD, standard deviation.

In 37.6% ± 4.3% (standard deviation) of samples isolated from *I. persulcatus* and in 3.6% ± 2.0% of samples from *D. reticulatus*, DNA of *B. burgdorferi* sensu lato complex was found (Table). The nucleotide sequences of the 5S-23S intergenic spacer (216–237 bp) determined in this study were compared to those of other *B. burgdorferi* sensu lato sequences. The sequences from *I. persulcatus* ticks were placed in 2 clades of monophyletic origin, which corresponded to *B. garinii* and *B. afzelii* with excellent bootstrap support (99% and 100%, respectively), whereas samples from *D. reticulatus* were more closely related to *B. garinii* (Figure 2). Thirty-four PCR-positive samples contained DNA of *B. garinii* (23 samples of *B. garinii* group NT29 and 11 of *B. garinii* group 20047), and 11 samples contained *B. afzelii* DNA (Table). For 2 PCR-positive samples from *I. persulcatus*, the hydrolysis of the PCR products with the *Tru9*I restriction endonuclease resulted in 6 fragments of 108, 68, 57, 50, 38, and 20 bp that corresponded to a mixture of patterns C and D (*27*) and, consequently, 2 species, *B. garinii* group NT29 and *B. afzelii* (Table). Among samples obtained from *D. reticulatus* ticks, 3 contained *B. garinii* group NT29 DNA, but no other variants were found.

*Anaplasma*/*Ehrlichia* DNA was found in 14 *I. persulcatus* ticks but not in *D. reticulatus* ticks from different areas of Novosibirsk region. PCR with primers specific to *A. phagocytophilum* 16S rRNA gene showed the human pathogen DNA in 3 samples, Ip-4, Ip-45, and Ip-68, collected from different areas of Novosibirsk region. The nucleotide sequences of 629 bp of all these samples were identical to each other (GenBank accession no. AY587607) and to the known *A. phagocytophilum* sequence (AF205140). Nucleotide sequences from 11 other DNA samples were identical to each other (GenBank accession no. AY587608) and differed from *E. muris* DNA sequence (U15527) at the single position 91 (C→T). In a phylogenetic tree created by the UPGMA method, both *A. phagocytophilum* and *E. muris* sequences evidently formed the distinctive clusters (Figure 3).

*Bartonella* DNA was detected by using nested PCR with primers that corresponded to the *groEL* gene in 47 *I. persulcatus* and 18 *D. reticulatus* ticks (Table). Comparative analysis of the *groEL* gene fragment nucleotide sequences of 190 bp showed 2 species, *B. henselae* and *B. quintana*, in both tick species. Part of the data is shown in Figure 4. The evidently separated 2 clades, *B. henselae* and *B. quintana*, were monophyletic with good statistical support (99% and 90%, respectively).

*Babesia* DNA was found in 3 *D. reticulatus* ticks (Dr-2, Dr-4, Dr-5) by nested PCR and was not detected among *I. persulcatus* studied (Table). The nucleotide sequences of the *Babesia* 18S rRNA gene fragment of 1,203 bp determined in this study were similar to each other and to the single known full-length *B. canis canis* nucleotide sequence (GenBank accession no. AY072926). In the phylogenetic tree, nucleotide sequences from Dr-2 and Dr-5 as well as the *B. canis canis* sequence formed a distinctive cluster that was separated from other *B. canis* subspecies with excellent bootstrap support (Figure 5). Direct sequencing of the PCR fragment from the tick Dr-4 showed a mixture of nucleotide sequences with 2 undetermined bases at positions 609 and 610. Diluting DNA 10 times allowed us to determine 2 nucleotide sequences. The first was identical to those from Dr-2 and the second to a *B. canis canis* sequence found in canine blood from Croatia (AY072926).

UPGMA analysis produced phylogenetic trees (Figures 2–5) that were almost identical to the neighbor-joining trees and results of phylogenetic analysis with maximal parsimony and minimal evolution approaches (trees not shown).

## Discussion

*I. persulcatus* is believed to maintain spirochetes transtadially and to transmit *Borrelia* to animals (*31*). Previously, the spirochetelike cells were isolated from *I. persulcatus* in Barbour-Stoenner-Kelly-H cultural medium (*2*) and were observed by indirect immunofluorescence assay (*24*). The nested PCR with subsequent sequencing showed that *I. persulcatus* contained both *B. afzelii* and *B. garinii* DNA (Table) as was previously shown (*2**,**10**,**32**,**33*). *B. garinii* appeared to be the prevalent species in *I. persulcatus* in Western Siberia (*33*). The *B. garinii* NT29 group is widely spread not only in Western Siberia but in the Russian Far East (GenBank accession no. AY429014, AY429015), Japan (*34**,**35*), and China (*36*). The nested PCR with subsequent sequencing allowed us to detect DNA of *B. garinii* group NT29 in 3.6% ± 2.0% of *D. reticulatus* ticks. Although *Borrelia*-specific DNA was detected in samples from *D. reticulatus*, numerous previous attempts to cultivate the living spirochetes were unsuccessful (*2*). Therefore, the ability of *D. reticulatus* to transmit *Borrelia* spp. remains unknown.

*E. muris* was the prevalent species among *Anaplasma*/*Ehrlichia* and was found in 8.8% ± 2.5% of *I. persulcatus* ticks in Western Siberia. This finding coincided with the *E. muris* prevalence (3%–13%) described in Baltic regions of Russia (*12*) and Siberia (*16*). The infection rate of *I. persulcatus* ticks with the human pathogen *A. phagocytophilum* (2.4% ± 1.4%) was significantly lower than the rate of infection with *E. muris*. In other regions, the infection rate of *I. persulcatus* with *A. phagocytophilum* varied from 1% to 4% in both China and Russia (*14**–**16**,**37*). The comparison of the *Anaplasma* 16S rRNA gene fragment nucleotide sequences (Figure 3) showed several genovariants of *A. phagocytophilum*. In *I. persulcatus* ticks, 4 types of sequences were found: 3 in China (GenBank accession nos. AY079425, AF205140, AF227954) and 1 in Korea (AF470701). All 3 nucleotide sequences of *A. phagocytophilum* determined in this study coincided with 1 genovariant from China (AF205140) found only in *I. persulcatus* (Figure 3) but not with the *A. phagocytophilum* isolated earlier in West Ural, Russia (GenBank accession no. AY094353) (*38*). No correlation was seen between genovariant and specific host or location.

Several tick species, such as deer ticks, *I. persulcatus*, *I. ricinus*, and *I. pacificus*, have been found to harbor *Bartonella* spp (*17**–**20*). Thus, PCR with primers specific to the 16S rRNA gene has shown *Bartonella* DNA in >70% of *I. ricinus* ticks in the Netherlands (*18*). Different *Bartonella* species, including *B. henselae*, have been detected in 19.2% of questing *I. pacificus* ticks in California by amplifying and sequencing the gltA gene fragment (*17*). More recently, *B. henselae* DNA has been found in 1.5% of *I. ricinus* ticks removed from humans in northwestern Italy (*19*) and in 38%–44% of *I. persulcatus* in Western Siberia (*20*). The high *Bartonella* infection rate of *I. persulcatus* in Western Siberia in 2003 and 2004 coincided with our observations from previous years (*20*). Moreover, both *B. henselae* and *B. quintana* were found not only in the 2 tick species studied (Figure 4) but also in *Aedes* mosquitos (O. Morozova, unpub. data). Only 2 human pathogens, *B. henselae* and *B. quintana*, were found in ixodid ticks in Siberia, despite sample collection for 4 years and phylogenetic analysis of all known *Bartonella* species (Figure 4).

We did not detect *Babesia* spp. in *I. persulcatus*. The only species of *Babesia* detected in *D. reticulatus* was *B. canis canis*, which causes babesiosis in dogs (*7*). *D. reticulatus* is the only known vector for *B. canis canis* (*23**,**39*). Comparison of the previously known *Babesia canis canis* 18S rRNA gene nucleotide sequences showed 3 genetic variants of *B. canis canis* in canine blood from Europe that differed at 2 variable positions 609 and 610 (*26**,**40*). Two of these variants were also seen in ticks in Novosibirsk. A new *B. canis canis* genetic variant that differed in a single nucleotide transition from those previously described was found. To our knowledge, this report is the first to identify nucleotide sequences of *B. canis canis* in ticks. *B. microti* was not found among tick samples studied, despite the presence of this human pathogen in small mammals in the same area (V. Rar, unpub. data).

When the 2 tick species were compared, *I. persulcatus* was more likely than *D. reticulatus* to be the host for tickborne bacterial infections examined in Western Siberia, Russia. The *Borrelia*, *Anaplasma*/*Ehrlichia*, and *Bartonella* infection rates for *I. persulcatus* exceeded those for *D. reticulatus* (Table). Moreover, *Borrelia* (*10**,**33*) and *Bartonella* (*20*) DNA from *I. persulcatus* could be easily detected in a single PCR, whereas nested PCR was required to detect DNA in samples from *D. reticulatus*. Neither *Anaplasma* nor *Ehrlichia* spp. were found in *D. reticulatus*. Conversely, *Babesia* spp. were detected only in *D. reticulatus*. The infection of unfed adult *I. persulcatus* and *D. reticulatus* ticks reflected transtadial transmission of tickborne infectious agents.

The experimentally observed and theoretically expected values of mixed infections of ticks with *Borrelia*, *Ehrlichia*, and *Bartonella* were statistically similar and consistent with independent distribution of these pathogens as previously reported (*10*). Thus, simultaneous coinfection with *Borrelia*, *Anaplasma*/*Ehrlichia*, and *Bartonella* found in 2.9% of *I. persulcatus* ticks slightly exceeded statistical probability of 1.8%. Further studies are required to establish the role of different tick species and biting arthropods as natural vectors of bacterial and protozoan agents.
